# Apoplastomes of contrasting cacao genotypes to witches’ broom disease reveals differential accumulation of PR proteins

**DOI:** 10.3389/fpls.2024.1387153

**Published:** 2024-05-16

**Authors:** Ivina Barbosa De Oliveira, Saline dos Santos Alves, Monaliza Macêdo Ferreira, Ariana Silva Santos, Keilane Silva Farias, Elza Thaynara Cardoso de Menezes Assis, Irma Yuliana Mora-Ocampo, Jonathan Javier Mucherino Muñoz, Eduardo Almeida Costa, Karina Peres Gramacho, Carlos Priminho Pirovani

**Affiliations:** ^1^ Departamento de Ciências Biológicas (DCB), Centro de Biotecnologia e Genética (CBG), Universidade Estadual de Santa Cruz (UESC), Ilhéus, Bahia, Brazil; ^2^ Molecular Plant Pathology Laboratory, Centro de Pesquisa do Cacau (CEPEC/CEPLAC), Ilhéus, Bahia, Brazil

**Keywords:** apoplast, defense proteins, apoplastic washing fluid, *Theobroma cacao*, *Moniliophthora perniciosa*

## Abstract

Witches’ broom disease (WBD) affects cocoa trees (*Theobroma cacao* L.) and is caused by the fungus *Moniliophthora perniciosa* that grows in the apoplast in its biotrophic phase and later progresses into the tissues, causing serious losses in the production of cocoa beans. Therefore, the apoplast of *T. cacao* can provide important defense responses during the interaction with *M. perniciosa*. In this work, the protein profile of the apoplast of the *T. cacao* genotypes Catongo, susceptible to WBD, and CCN-51, resistant one, was evaluated. The leaves of *T. cacao* were collected from asymptomatic plants grown in a greenhouse (GH) and from green witches’ brooms grown under field (FD) conditions for extraction of apoplastic washing fluid (AWF). AWF was used in proteomic and enzymatic analysis. A total of 14 proteins were identified in Catongo GH and six in Catongo FD, with two proteins being common, one up-accumulated, and one down-accumulated. In CCN-51, 19 proteins were identified in the GH condition and 13 in FD, with seven proteins being common, one up-accumulated, and six down-accumulated. Most proteins are related to defense and stress in both genotypes, with emphasis on pathogenesis-related proteins (PR): PR-2 (β-1,3-glucanases), PR-3 and PR-4 (chitinases), PR-5 (thaumatine), PR-9 (peroxidases), and PR-14 (lipid transfer proteins). Furthermore, proteins from microorganisms were detected in the AWF. The enzymatic activities of PR-3 showed a significant increase (p < 0.05) in Catongo GH and PR-2 activity (p < 0.01) in CCN-51 FD. The protein profile of the *T. cacao* apoplastome offers insight into the defense dynamics that occur in the interaction with the fungus *M. perniciosa* and offers new insights in exploring future WBD control strategies.

## 1 Introduction

The species *Theobroma cacao* originated in South America (Motamayor et al., 2013). This species produces cocoa, which plays a very important role in the global economy, especially in the chocolate industry, because its beans are the main raw material for chocolate and its derivatives (Beg et al., 2017). According to reports from the International Cocoa Organization (ICCO, 2022), global cocoa bean production was approximately 5.2 million tons in 2021. The African continent stood out by contributing the largest share, 77% of the total production. In this scenario, Brazil is one of the main producers in the Americas, totaling almost 300 thousand tons in 2023 (IBGE, 2024).

Cocoa farming is threatened by one of the most serious agricultural diseases, witches’ broom disease (WBD), caused by the hemibiotrophic fungus *Moniliophthora perniciosa* (Aime and Phillips-Mora, 2005; Meinhardt et al., 2008; Evans, 2016; Santos et al., 2023). WBD can cause substantial losses to cocoa farming, with particular impact on plantations in Brazil, specifically in the state of Bahia, which has been facing challenges since the arrival of the fungus in 1989 (Santos et al., 2023). During the biotrophic phase, the fungus *M. perniciosa* grows in the apoplast of *T. cacao*, which represents a key environment in the interaction between plant molecules and molecules released by *M. perniciosa* (Ceita et al., 2007; Meinhardt et al., 2008; Santos et al., 2023).

The apoplast is an essential compartment in various physiological processes and in the plant communication with the environment. Its relevance is evident because it influences important responses to environmental stresses and plays a crucial role in plant defense against pathogens (Sakurai, 1998; Agrawal et al., 2010). Communication between apoplast with the environment is fundamental for the activation of defense mechanisms, contributing to the plant ability to quickly recognize and respond to pathogenic threats, thus strengthening resistance against invasive molecules (Agrawal et al., 2010; Doehlemann and Hemetsberger, 2013).

The apoplastic protein content of the plant is modified during plant–pathogen interaction (Martínez-González et al., 2018). Many studies have aimed to understand this modulation in order to gain better knowledge of the behavior of both the pathogen, by interfering in the plant’s defense, and of the plant whose normal development is impaired (Martínez-González et al., 2018; Wang and Wang, 2018; Hassett et al., 2020). The advent of omics technologies, particularly proteomics, has helped to elucidate cellular responses involved in different biological conditions (Mares et al., 2016, Mares et al., 2017, Mares et al., 2020; de Almeida et al., 2023; de Oliveira et al., 2024; Zugaib et al., 2023).

Understanding the molecular processes involved in the apoplast during a plant–pathogen interaction remains a challenge. Apoplastic proteomic studies, even with their challenges, have contributed to the understanding of molecular processes in pathogen–host interactions. Many defense proteins, such as pathogenesis-related proteins (PR proteins), have been identified in the apoplast (Floerl et al., 2008; Guerra-Guimarães et al., 2015; Li et al., 2016). Recently, the first apoplastic proteomic profile of contrasting genotypes regarding *Theobroma cacao* resistance to the WBD was reported (de Oliveira et al., 2024).

The term apoplastome described by de Oliveira et al. (2024) refers to the set of proteins identified in the apoplast of contrasting *T. cacao* genotypes regarding resistance to WBD. The proteins identified in this study are mainly involved in defensive processes of *T. cacao*, such as peroxidases, chitinases, and osmotin. Furthermore, the efficiency of the apoplastic washing fluid (AWF) from *T. cacao* genotypes in inhibiting the germination of *M. perniciosa* spores was demonstrated. The inhibition reached almost 90% for the resistant genotype (CCN-51) and 82% for the susceptible one (Catongo), as well as inducing morphological alterations in basidiospores (de Oliveira et al., 2024). These results reveal that the *T. cacao* apoplast represents a fundamental compartment in the complex defense network of plants.

Considering this, the present work aims to characterize the protein profile of the AWF obtained from asymptomatic plants grown in a greenhouse (GH) and from broom branches collected from field (FD)–grown plants with WBD, in two contrasting genotypes for WBD resistance (Catongo-susceptible and CCN-51–resistant). Our intention is to deepen the understanding of *T. cacao* defense responses, thus comprehending the role of the apoplast in the *T. cacao–M. perniciosa* interaction, to contribute to disease control efforts in the future.

## 2 Materials and methods

### 2.1 Collection of biological material and extraction of apoplastic fluid

Fully expanded mature leaves of adult *T.cacao* plants of the genotypes Catongo (susceptible to *M. perniciosa*) and CCN-51 (resistant to *M. perniciosa*) (Gramacho et al., 1992) cultivated under FD and GH conditions were collected and used to extract AWF. The collection of mature leaves on branches infected by *M. perniciosa* (green witches’ broom) were carried out randomly at the Active Germplasm Bank (BAG) of CEPLAC/CEPEC in Ilhéus, Bahia, Brazil (14°45′40.2″S, 39°14′03.9″W) on plants under FD conditions. Leaves from asymptomatic plants, grown in a GH at CEPLAC/CEPEC, were also used. AWF extraction was performed by vacuum infiltration followed by centrifugation, as previously described (Pirovani et al., 2008; de Oliveira et al., 2024). A total of approximately 200 μL/g fresh weight of *T. cacao* leaf was collected. For proteomic analysis, approximately 1,200 g of leaves of the Catongo GH genotype, 715 g of Catongo FD leaves, 800 g of CCN-51 GH leaves, and 750 g of CCN-51 FD leaves were collected. For the analysis of chitinase, β-1,3-glucanase, and protease activity, three biological replicates were performed for each sample using a total of five leaves in each AWF extraction.

### 2.2 Protein extraction from apoplastic fluid and SDS-PAGE

The collected AWF was lyophilized in aliquots with a volume of 15 mL and used in protein extraction according to Wang et al. (2003), with modifications according to Pirovani et al. (2008). After protein recovery, the precipitate was resuspended in urea (8 mol L^−1^) and quantified using the 2-D Quant Kit (Cytiva), according to the manufacturer’s recommendations, using BSA (bovine serum albumin) as standard.

After quantification, the proteins were resolved in SDS-PAGE (sodium dodecyl sulfate–polyacrylamide gel electrophoresis) in mini vats electrophoresis (Hoefer), with 8 cm × 10 cm gels at 12.5% acrylamide (Laemmli, 1970). A total of 30 μg of AWF protein from *T. cacao* genotypes was applied onto gels, along with 30 μg of total leaf protein extract (TE) from CCN-51 FD extracted according to Pirovani et al. (2008), to verify the protein band profiles of each sample (AWF and TE) and their differences.

### 2.3 Mass spectrometry and database analysis

A mass of 100 µg of AWF proteins from *T. cacao* genotype samples was treated and subjected to tryptic digestion according to Villén and Gygi (2008) with modifications described by de Almeida et al. (2023). The peptides were then analyzed in technical triplicates on a liquid chromatography system (Agilent 1290 Infinity II HPLC) coupled to a quadrupole/Time-of-Flight mass spectrometer (Agilent 6545 LC/QTOF) with C18 columns (100 mm × 100 mm) (de Almeida et al., 2023).

To analyze the spectral data generated for the identification of peptides, we used the software Spectrum Mill from the Broad Institute (Rev BI.07.08.214). Raw data files were converted to a peak list format (mgf) without summing the scans by the Mascot Distiller software v.2.6.2.0, 2009 (Matrix Science Ldt.). After extracting the mass spectrometry (MS)/MS spectra, a database search of *T. cacao* and fungi was performed. All databases were downloaded from UniProt (https://www.uniprot.org). A database of the theoretical *T. cacao* apoplastome (de Oliveira et al., 2024) was also used to identify predicted proteins for the apoplast. The research was carried out according to the following configurations: carbamidomethylation with fixed modifications, methionine oxidation with variable modification, trypsin for cleavage, and 0.1-Da tolerance for precursor and fragment ions. Statistical analyses were performed using the Mass Profiler Professional 15.1 software (MPP; Agilent), determined through analyses in which the inclusion criteria were defined as p ≤ 0.05 and a fold change ≥ 1.5.

The identified proteins were submitted for functional annotation and categorization according to function and cellular processes using the BLAST2GO software (Conesa et al., 2005) available at the OmicsBox platform (https://www.biobam.com/blast2go/).

### 2.4 Comparison of methods for characterizing the *T. cacao* apoplastome

The extraction efficiency of AWF from *T. cacao* leaves for recovery of apoplastic proteins was compared and evaluated with the efficiency of total extract (TE) of *T. cacao* leaves for identification of apoplastic proteins.

Initially, proteins were recovered from the AWF of *T. cacao* leaves carried out in the present study obtained as described in the section “Protein extraction from apoplastic fluid and SDS-PAGE.” Then, files containing raw peptide data from TE samples from *T. cacao* leaves carried out in previous work (de Almeida et al., 2023) were used for comparison. In terms of methodology comparison, only the CCN-51 variety was used. The raw peptide data were analyzed against the *T. cacao* database and the database of predicted proteins for the apoplast (de Oliveira et al., 2024) as per the previous section (“Mass spectrometry and database analysis”). For this analysis, differences in accumulation among the identified proteins were not taken into account. The data obtained did not need to pass statistical inclusion criteria (p ≤ 0.05 and fold change ≥ 1.5). Verification was based on the number of proteins obtained in each sample rather than on protein abundance.

### 2.5 Transcript profile of predicted apoplast proteins

The transcript pattern for the predicted apoplast proteins was verified from reference files containing transcripts from *Theobroma* cacao cv. ‘Comum’ (Forastero). The files in FASTA format (GCA_000208745.2_Criollo_cocoa_genome_V2/and GCA_000403535.1_Theobroma_cacaoMatina) were obtained from the GenBank database (https://ftp.ncbi.nlm.nih.gov/genomes/genbank/plant/Theobroma_cacao/latest_assembly_versions/). Relative quantification was performed for the six transcripts exclusive to the Catongo genotype and 19 exclusive to CCN-51, with 14 being common, totaling 39 transcripts, which correspond to the diversity of proteins identified in the apoplast of *T. cacao* in this study. Therefore, proteins that appeared repeatedly in the samples of both Catongo and CCN-51, regardless of the growth condition, were excluded to prevent duplication. Public data were used from10 *T. Cacao* RNA-Seq libraries (Teixeira et al., 2014), available at NCBI’s Sequence Read Archive (SRA) (https://www.ncbi.nlm.nih.gov/sra) under accession number SRA066232, with five libraries of the control condition and five of the *M. perniciosa*–infected condition. The relative quantification of *T. cacao* transcripts and ranking in descending order of expression was performed with the Galaxy platform (https://rna.usegalaxy.eu), using the Salmon extension (Patro et al., 2017). The corresponding proteins of each analyzed transcript were found with the BlastX search tool (https://blast.ncbi.nlm.nih.gov/).

To visualize the expression profile of the transcripts, a heatmap was plotted using the Complex Heatmap packages in the statistical software R (R Core Team, 2022).

### 2.6 System biology analysis

The protein–protein interaction network was created with the proteins identified in the apoplast of the Catongo and CCN-51 genotypes of *T. cacao* using the homologous sequences of *Arabidopsis thaliana* as reference. The network was built according to the STRING 11.5 database (https://string-db.org/) based on the following parameters: high level of confidence (0.7), no more than 50 interactions, and addition of nodes until network saturation.

The results obtained were analyzed using the Cytoscape software version 3.9.1 (https://cytoscape.org/) (Shannon et al., 2003). The Igraph package of the statistical tool R Studio was used to group the proteins and calculate the centrality (betweenness) and nodes (degree) parameters. To enrich genetic ontology terms and categories, the Biological Networks Gene Ontology v.3.0.3 (BiNGO) tool was used (Maere et al., 2005) with a Cytoscape plugin (https://apps.cytoscape.org/apps/bingo).

### 2.7 Western blot

A mass of 30 μg of AWF protein from cacao genotypes and 7 μL of molecular weight marker (Kaleidoscope, pre-stained, 0.45 μm pore, Bio-Rad, USA) were applied to SDS-PAGE in electrophoresis minivats (Hoefer), with 8 cm × 10 cm gels containing 12.5% acrylamide and run at a constant amperage of 30 mA as described by Oliveira et al. (2020). The membranes were incubated with PR-3 primary antibodies (8:20,000) for 1 h. Subsequently, the membrane was washed three times, for 15 min each wash, with 1× Tris-buffered saline (TBS-T) buffer followed by incubation with the secondary antibody (anti-rabbit Immunoglobulin G (IgG)) conjugated to alkaline phosphatase (7.5 μ:2000 of 1× TBS-T) for 2 h. After incubation, the membrane was washed again three times and finally incubated for 15 min with development buffer [MgCl_2_, 5 mmol L^−1^; NaCl, 100 mmol L^−1^; Tris-HCl (pH 9.8), 100 mmol L^−1^; and H_2_O]. The development was carried out in the absence of light with Nitroblue tetrazolium/5-bromo-4-chloro-3-indolyl phosphate (NBT/BCIP) substrates for approximately 5 min. Finally, the apparent accumulation was determined from images of the nitrocellulose membrane, using the Gel Quant Net 1.8.2 program (http://biochemlabsolutions.com/GelQuantNET.html).

### 2.8 Enzyme activities

For enzyme activity analysis, AWF from Catongo and CCN-51 GH and FD genotypes was collected by infiltration in ice-cold distilled water as described by de Oliveira et al. (2024). A 30-μL aliquot of each sample was separated for quantification by the Bradford method (Bradford, 1976) using BSA as standard.

#### 2.8.1 Chitinase activity

For the analysis of chitinase activity, the Chitinase Assay Kit, Fluorimetric (SIGMA-ALDRICH), was used according to the manufacturer’s recommendations. The assay was based on the enzymatic hydrolysis of chitinase substrates that releases 4-methylumbelliferone (4MU). The fluorescence released in basic medium was measured at an excitation wavelength of 360 nm and an emission wavelength of 450 nm. Samples were pipetted in duplicates into 96-well microplates and analyzed on a SpectraMax Paradigm (Molecular Devices). Two substrates were used in each test: the substrate 4-methylumbelliferyl beta-D-N,N′,N″-triacetylchitotriose (for detection of endochitinase activity) and methylumbelliferyl beta-D-N,N′-dediacetylchitobioside hydrate (for detection of exochitinase activity). In each assay, chitinase from *Trichoderma viride* was used as positive control and the blank (only the substrate) as negative control. The calculation of chitinase activity was performed according to the standard curve. One unit of chitinase activity was defined as the amount of enzyme to release 1 μmol of 4-metilumbeliferona per minute.

#### 2.8.2 β-1,3-glucanase activity

The enzymatic activity of glucanase was determined in accordance with the 3,5-dinitrosalicylic acid (DNS) method for measuring the released reducing sugar, as reported by Miller (1959). A reaction solution containing laminarin as substrate (2 mg mL^−1^) and crude AWF samples from *T. cacao* genotypes, in a 1:1 ratio, was incubated at 40°C under shaking for 18 h. The samples were then centrifuged at 1,792 g for 5 min and the supernatant was collected. Next, 200 μL of the reaction solution and 100 μL of the DNS solution were added to new microtubes, which were vortexed and incubated in a water bath at 100°C for 5 min. Then, the reaction was stopped by cooling on ice for about 2 min. The final volume was adjusted to 1,000 μL by adding water, and, finally, the samples were pipetted in quadruplicate into 96-well microplates and analyzed in a SpectraMax Paradigm multi-mode microplate reader (Molecular Devices), with absorbance read at a wavelength of 550 nm. Controls of each sample containing laminarin, AWF sample, and DNS were also read. A glucose standard curve was plotted as a reference. One unit (U) of enzyme activity was defined as the amount of enzyme required to release 1 μmol of glucose per minute.

#### 2.8.3 Protease activity

The AWF of *T. cacao* genotypes was suspended in non-reducing loading buffer [50% glycerol, Tris-HCl (pH 6.8), and 1% bromophenol blue], and the samples were applied to 10% SDS-PAGE (Michaud et al., 1996; Pirovani et al., 2010). After the run, the qualitative analysis of the proteases present in the samples was carried out using 0.1% gelatin gel and stained in colloidal Coomassie G-250 (0.08%) according to Neuhoff et al. (1988).

### 3 Results

#### 3.1 Identification of AWF proteins from *T. cacao* genotypes

**Figure 1:**
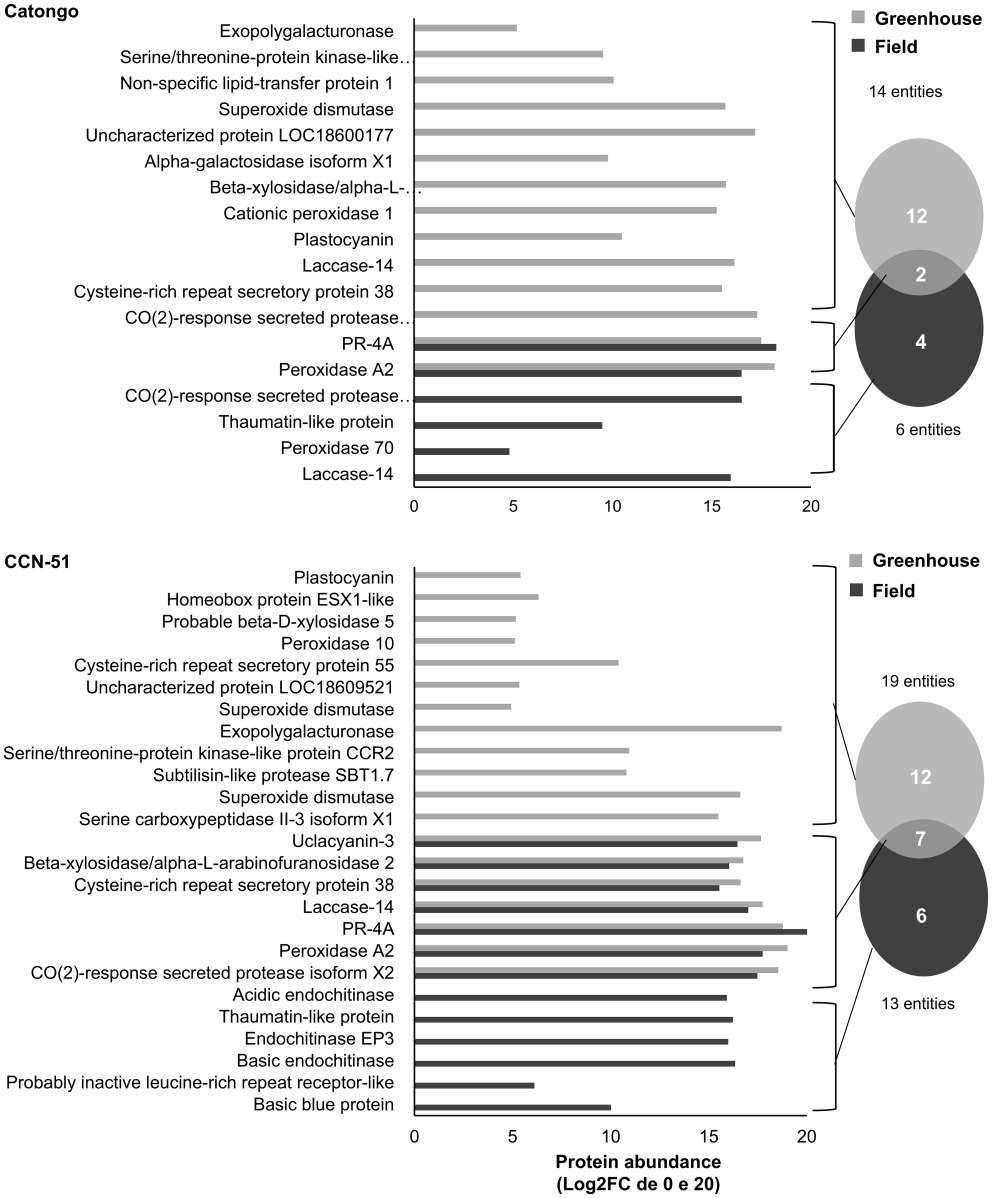
Proteins identified in the apoplastic washing fluid within *T. cacao* genotypes under different growth conditions. Unique and differentially abundant proteins (p < 0.05 and fold change ≥ 1.5) identified in the apoplastic fluid of the *T. cacao* Catongo (susceptible) and CCN-51 (resistant) genotypes of asymptomatic plants grown in a greenhouse and plants at the stage of green witch’s broom grown in field conditions.

**Figure 2:**
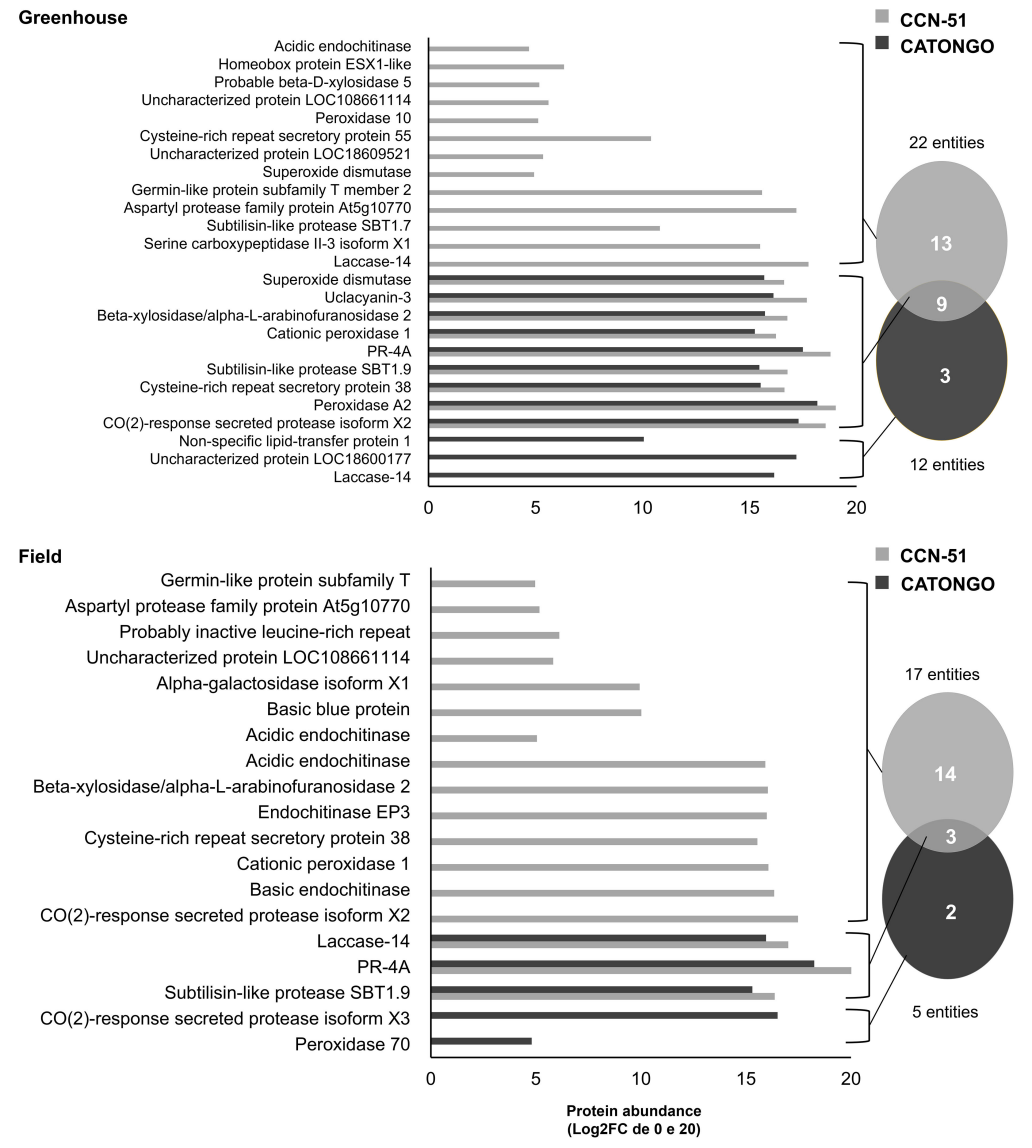
Proteins identified in the apoplastic washing fluid between *T. cacao* genotypes under different growth conditions. Unique and differentially abundant proteins (p < 0.05 and fold change ≥ 1.5) identified in the apoplastic fluid of the *T. cacao* Catongo (susceptible) and CCN-51 (resistant) genotypes of asymptomatic plants grown in a greenhouse and plants at the stage of green witch’s broom grown in field conditions.

The AWF proteins were resolved on SDS-PAGE for qualitative analysis alongside the total leaf protein extract (TE) samples to confirm differential bands between AWF and TE protein samples (**Supplementary Figure 1**). The full identification of *T. cacao* AWF peptides was initially carried out using the general *T. cacao* database (**Supplementary Figure 2**). Next, the AWF peptides were also identified against the library of proteins predicted for the apoplast proposed by de Oliveira et al. (2024) considering differentially abundant proteins (p ≤ 0.05 and a fold change ≥ 1.5) and identified exclusively in both genotypes (**Figures 1**, **2**). For the Catongo genotype, 14 proteins were identified in the GH condition and six in the FD condition. Catongo presented 12 exclusive proteins in the GH condition and four exclusive ones in the FD condition, with two proteins in common, one up-accumulated (PR-4), and the other down-accumulated (Peroxidase A2) (**Figure 1**). For the CCN-51 genotype, 19 proteins were identified in the GH condition and 13 in FD. CCN-51 GH presented 12 unique proteins and six in the FD condition. There were seven proteins in common for both conditions, with one up-accumulated (PR-4) and six down-accumulated [Peroxidase A2, CO(2)-response secreted protease isoform X2, Laccase-14, Cysteine-rich repeat secretory protein, Beta-xylosidase/alpha-L-arabinofuranosidase 2, and Uclacyanin-3] (**Figure 1**).

In the comparison between the two genotypes in the GH condition, 22 proteins were identified in the resistant genotype (CCN-51) and 12 proteins in the susceptible genotype (Catongo), presenting 13 exclusive proteins in CCN-51 and three exclusive in Catongo. Nine proteins were in common, all being up-accumulated for the CCN-51 genotype [CO(2)-response secreted protease isoform X2, Peroxidase A2, Cysteine-rich repeat secretory protein, Subtilisin-like protease SBT1.9, PR-4, Cationic peroxidase, Beta-xylosidase/alpha-L-arabinofuranosidase 2, Uclacyanin-3, and Superoxide dismutase (SOD)] (**Figure 2**). In the FD condition, 17 proteins were identified in CCN-51 and five proteins in Catongo, with 14 exclusive to the CC51 genotype and two to the Catongo genotype. Three proteins were found in common to be up-accumulated in the resistant genotype (Subtilisin-like protease SBT1.9, PR-4, and Laccase-14) (**Figure 2**).

#### 3.2 Efficiency of AWF extraction for apoplastome characterization

**Figure 3:**
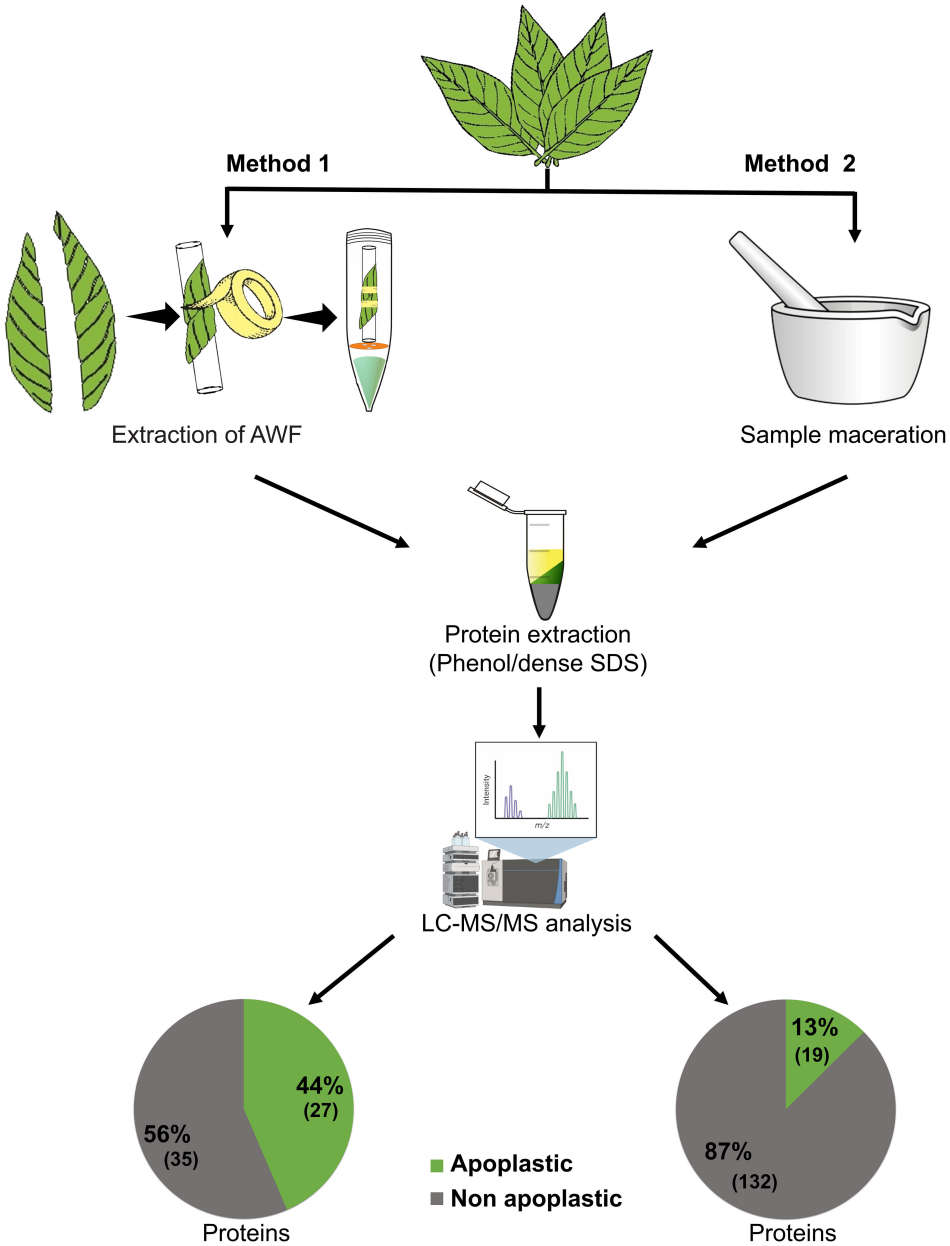
Efficiency of extracting apoplastic washing fluid from *T. cacao* leaves for characterization of the apoplastome compared to the total extract of *T. cacao* leaves. Method 1: Recovery of apoplastic proteins from the extraction of apoplastic washing fluid from leaves of the CCN-51 genotype (resistant), carried out in the present study. Method 2: Recovery of apoplastic proteins from the extraction of total extract from leaves of the CCN-51 genotype (resistant), carried out in a previous work (de Almeida et al., 2023). The raw peptide data were analyzed against the database of predicted proteins for the *T. cacao* apoplast (de Oliveira et al., 2024).

Raw peptide data from AWF samples (performed in the present study) and TE samples performed in previous work (de Almeida et al., 2023) of the CCN-51 genotype were initially analyzed against the *T. cacao* database to evaluate the efficiency of the methods used in the identification of apoplastic proteins. A total of 62 proteins were identified from AWF extraction and 151 proteins from TE (**Figure 3**). Next, the raw peptide data were analyzed against the database of predicted proteins for the *T. cacao* apoplast (de Oliveira et al., 2024). A total of 27 proteins (44%) predicted for the apoplast through AWF extraction and 19 proteins (13%) through leaf TE sampling (**Figure 3**).

#### 3.3 Transcriptional profile of proteins predicted for the apoplast of *T. cacao* genotypes

**Figure 4:**
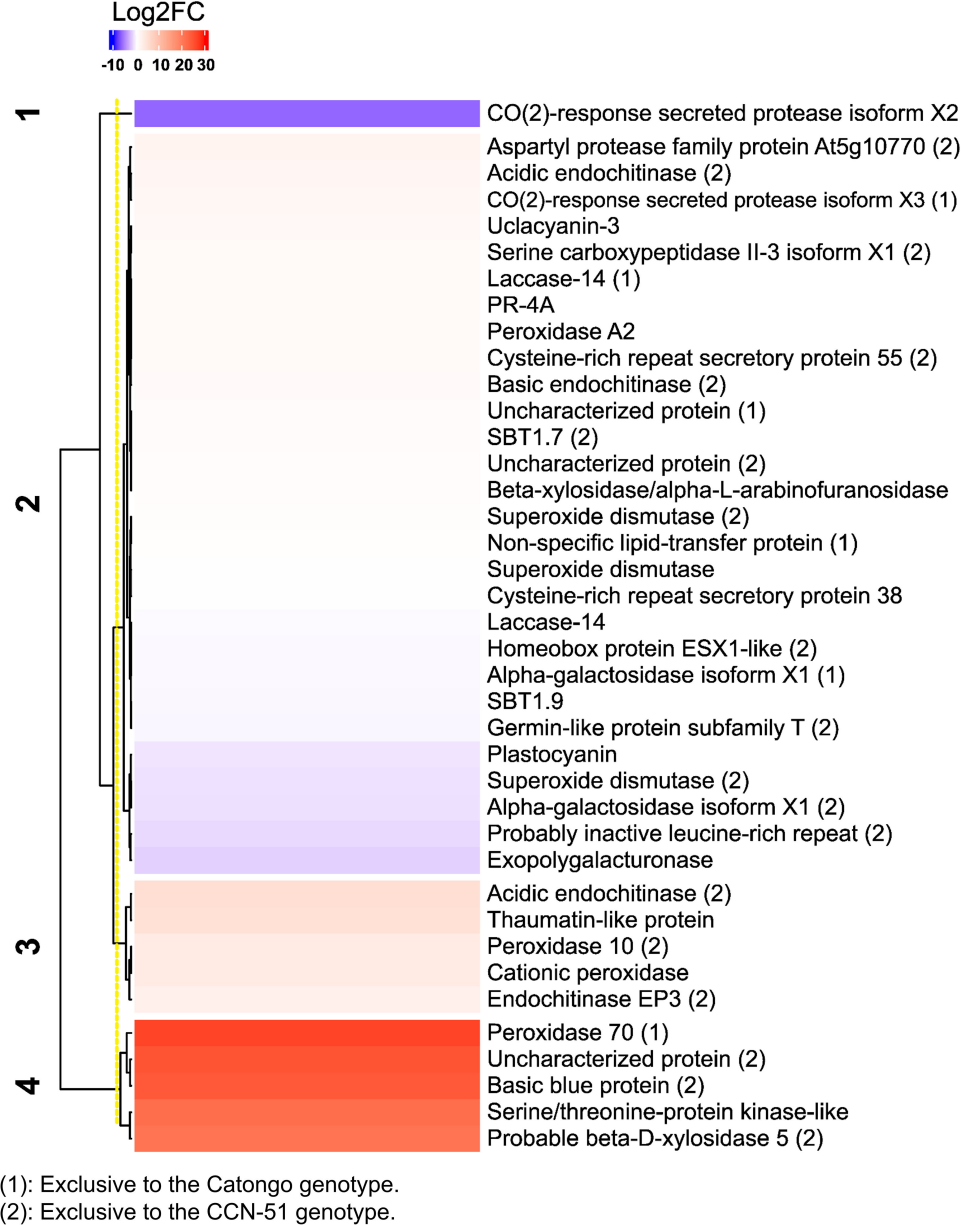
Transcriptional profile of apoplastic washing fluid proteins from *T. cacao* genotypes. Heatmap based on Pearson’s correlation coefficient, representing the differential accumulation of transcripts corresponding to apoplastic proteins of *T. cacao* genotypes in the database of Teixeira et al. (2014) (SRA066232). In blue, transcripts with reduced accumulation; in red, transcripts with relative increase in accumulation, related to the Log2FC scales of −10 and 30, respectively. The full name of the protein is described in **Supplementary Table 1**.

The majority transcripts were up-accumulated, such as the transcripts of the 70 peroxidase proteins identified in Catongo FD (lcl_NC_030853.1_mrna_XM_007033525.2_15125 with 89% coverage and 100% identity) and acidic proteins endochitinase and endochitinase EP3 identified in CCN-51 FD (lcl_NC _030850.1_mrna_XM_007051131.2_3841, with 74% coverage and 100% identity and lcl_NC_030853.1_mrna_XM_007033739.2_15288 with 78% coverage and 100% identity, respectively). A protein transcript common in both genotypes, thaumatin-like protein, also showed increased accumulation (lcl_NC_030852.1_mrna_XM_007040102.2_12731 with 69% coverage and 100% identity). On the other hand, transcripts referring to SOD proteins (lcl_NC_030854.1_mrna_XM_018122149.1_19942 with 54% coverage and 100% identity) identified in CCN-51 and CO(2)-response secreted protease isoform X2 identified in both genotypes (lcl_NC_030857.1_mrna_XM_007017808.2_26401 with 82% coverage and 100% identity) had reduced accumulation (**Figure 4**). All proteins showed coverage above 40% and 100% identity (**Supplementary Table 1**).

#### 3.4 Functional classification of *T. cacao* apoplastome proteins

**Figure 5:**
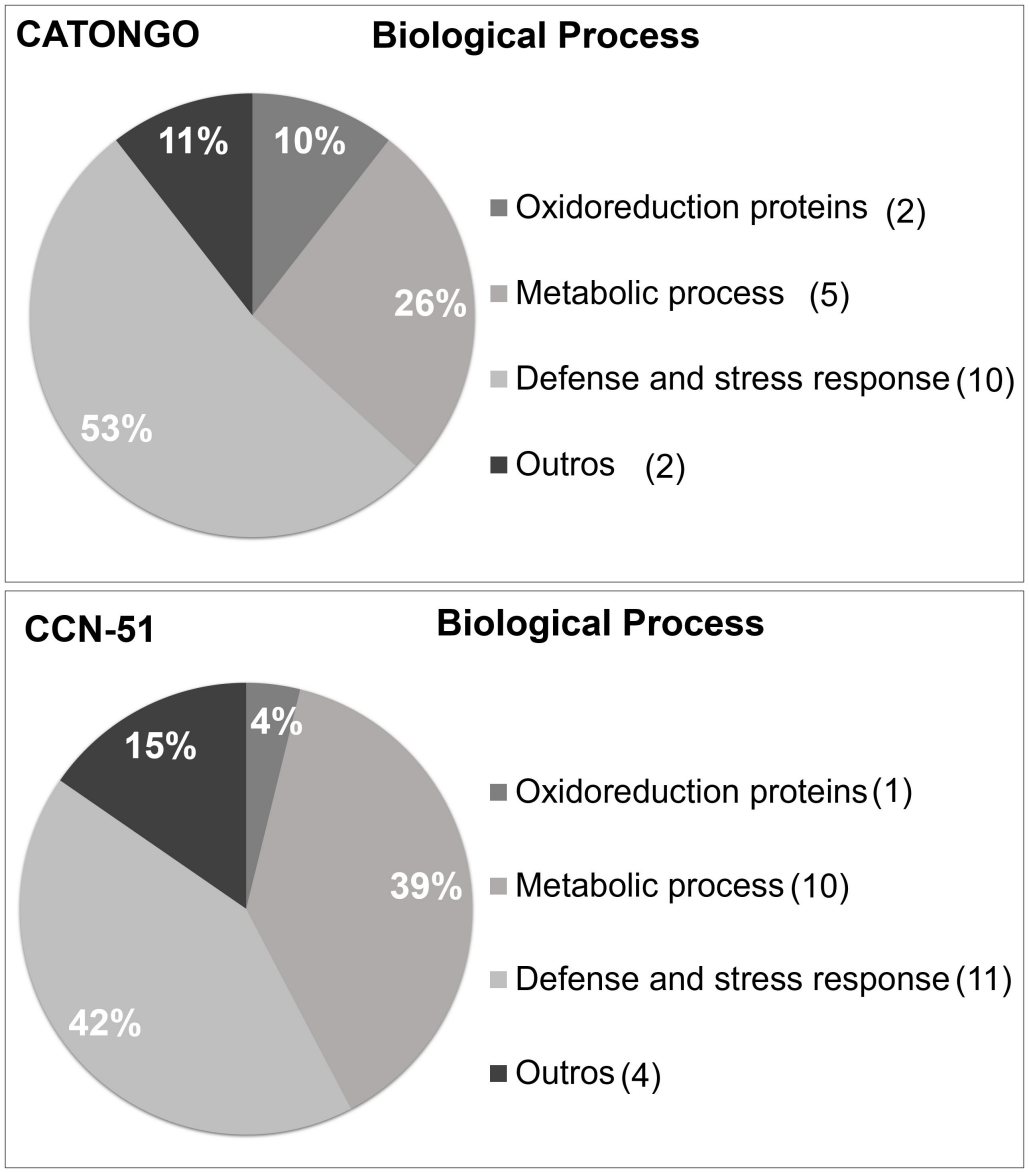
Functional annotation of proteins identified in apoplastic washing fluid from *T. cacao* genotypes. Characterization by biological processes of proteins identified in the apoplastic washing fluid of the *T. cacao* Catongo (susceptible) and CCN-51 (resistant) genotypes of asymptomatic plants grown in a greenhouse and plants at the stage of green witch’s broom grown in field conditions.

According to the functional classification, most of the proteins identified from both genotypes, regardless of the plant’s condition, were related to the defense and stress response and metabolic processes. For the Catongo genotype, 10 proteins (53%) were related to defense and 11 proteins (42%) were related to the CCN-51 genotype (**Figure 5**). A total of five proteins (26%) were involved in metabolic processes in Catongo and 10 proteins (39%) in CCN-51. Other categories were assigned in the functional classification, where one protein (4%) in the CCN-51 genotype was involved in redox processes and two (11%) proteins in the Catongo genotype.

#### 3.5 Interaction network of *T. cacao* apoplastome proteins

**Figure 6:**
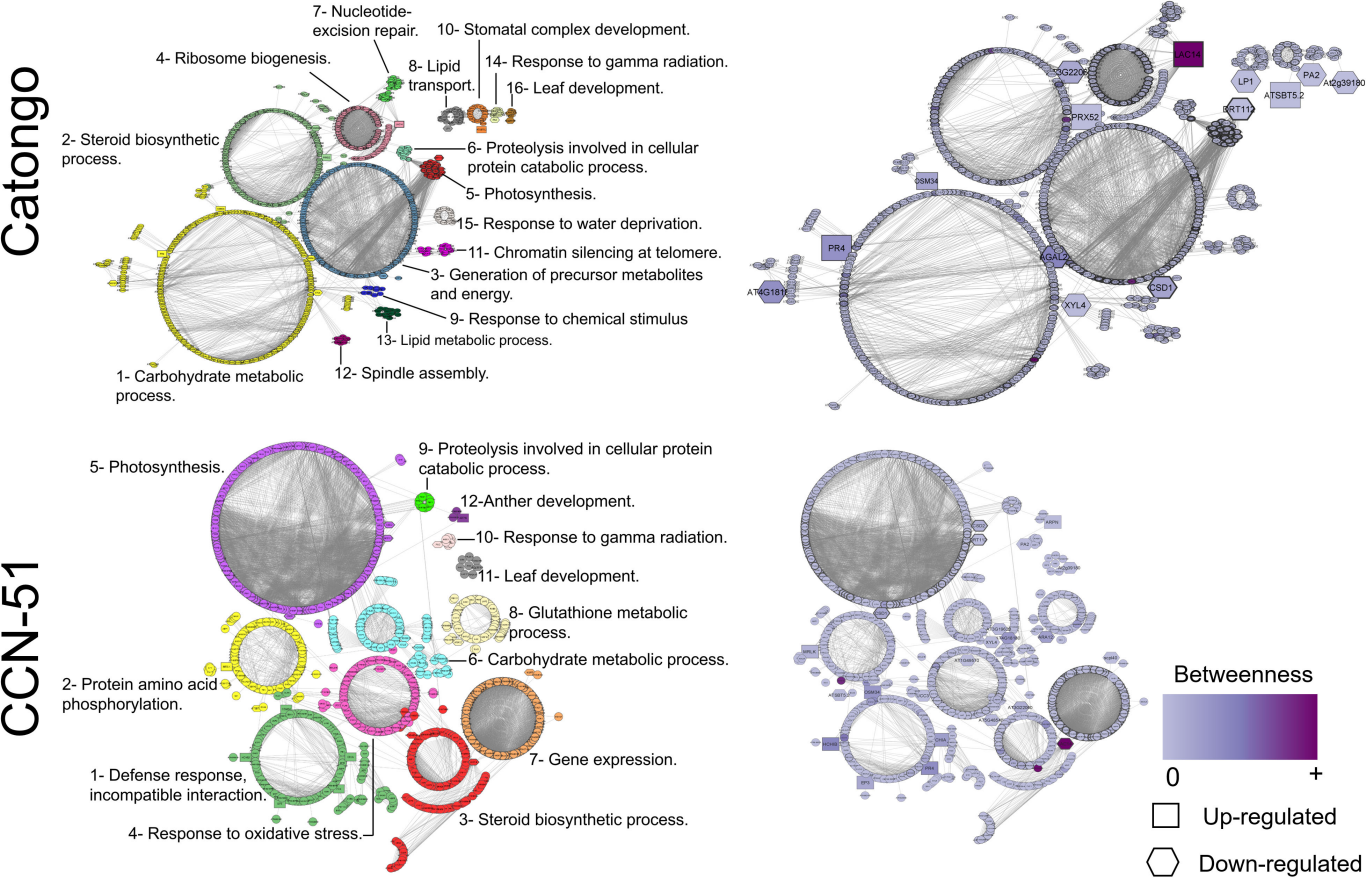
Protein–protein interaction network. Interaction network of apoplastic washing fluid proteins of the *T. cacao* Catongo (susceptible) and CCN-51 (resistant) genotypes according to homology with *Arabidopsis thaliana*. Each cluster is represented by a different color. The purple color scale represents the betweenness of a node and the thickness of each node represents the degree centrality. Interactions were generated and visualized in software STRING 10.5 and reconstructed by Cytoscape software.

The protein interaction network of the Catongo genotype samples (GH and FD) showed 722 nodes, which corresponded to each specific protein, and 5,718 connectors, which corresponded to physical or functional interactions between proteins. The CCN-51 genotype (GH and FD) resulted in 941 nodes and 7,251 connectors. The larger nodes correspond to proteins found within the network (**Figure 6**). Of the total of 20 orthologous protein sequences in the Catongo sample, 14 were found in the network. In CCN-51, out of 35 sequences, 23 were located within the network.

Based on the gene ontology for biological processes, 16 functional clusters for the Catongo genotype and 12 for the CCN-51 genotype were predicted. (**Figure 6**). According to the network analysis of the Catongo samples, the proteins predicted for cluster 1 stood out for being involved in the carbohydrate metabolic process. In cluster 1, there were 200 proteins, five of which were orthologous: Hevein-like preproprotein (PR4) and Osmotin-like (OSM34) that were up-accumulated and Alpha-galactosidase 2 (AGAL2) and Beta-D-xylosidase 4 (XYL4) down-accumulated. In cluster 2, the predicted proteins were involved in the steroid biosynthetic process, containing 117 proteins, two of which were orthologous, one peroxidase and one kinase (PRX52, up-accumulated, and AT3G22060, down-accumulated, respectively). Cluster 3 contained 119 proteins that may be involved in the generation of precursor metabolites and energy, with emphasis on the superoxide dismutase (CSD1) protein. In addition, cluster 4 contained 71 proteins possibly involved in ribosome biogenesis, with the only up-accumulated laccase (LAC14) orthologous protein (**Figure 6**).

The main clusters resulting from proteins of the CCN-51 genotype were cluster 1, where the proteins were involved in the incompatible interaction defense response. With 141 proteins, five of which were orthologous and were up-accumulated, the proteins basic endochitinase (HCHIB), osmotin (OSM34), chitinases (EP3 and CHIA), and hevein-like preproprotein (PR4). Cluster 3 with 130 proteins involved in the steroid biosynthetic process, having three orthologous proteins with reduced accumulation, the laccase proteins (LAC14) and two kinases (AT3G22060 and AT5G48540). Cluster 4 proteins may be involved in the response to oxidative stress, where it contains 89 proteins, two of which are up-accumulated orthologs, the uclacyanin protein (UCC3), and peroxidase (AT1G49570) (**Figure 6**).

The Laccase protein (LAC14), identified in both genotypes, presented a higher betweenness value (**Figure 6**), indicating a high interaction with other proteins (Nithya et al., 2023). The superoxide dismutase protein (CSD1) identified in both genotypes in the GH condition presented degree values above the average (**Figure 6**), being considered a protein that has an important regulatory role within the network (Nithya et al., 2023).

#### 3.6 Chitinase immunodetection

**Figure 7:**
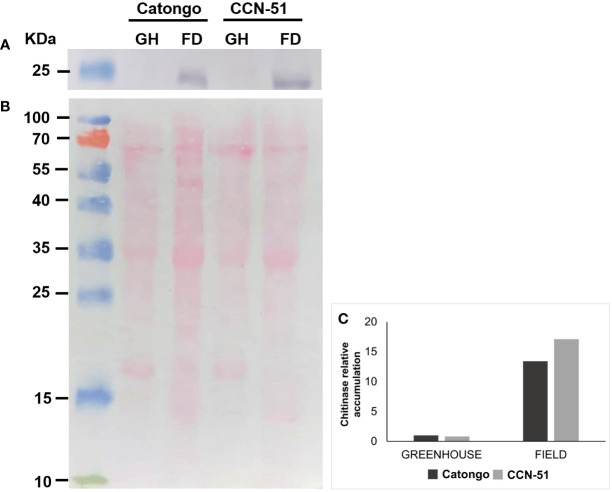
Immunodetection of PR-3 in apoplastic washing fluid from T. cacao genotypes. Western blot using an antibody against PR-3 in a protein sample from apoplastic washing fluid of T. cacao genotypes Catongo (susceptible) and CCN-51 (resistant) from asymptomatic plants grown in a greenhouse (GH) and of green witches’ broom stage plants grown under field (FD) conditions. **(A)** Protein accumulation in samples of apoplastic washing fluid from T. cacao genotypes. **(B)** Mirror gel. **(C)** Protein accumulation in T. cacao genotypes estimated by the Gel Quant Net 1.7.8 program. kDa, molecular weight.

The accumulation of chitinase (PR-3) identified in the apoplast of *T. cacao* genotypes was visualized by Western blot (**Supplementary Figure 3**; **Figure 7**). The presence of PR-3 was mainly detected in samples from plants of both genotypes under FD conditions (green witches’ broom stage) in both genotypes, with an increase of approximately 15 times in relation to samples in GH condition (asymptomatic grown in a GH) (**Figure 7**).

#### 3.7 Enzymatic activity of chitinase, β-1,3-glucanase, and proteases in AWF of *T. cacao*

**Figure 8:**
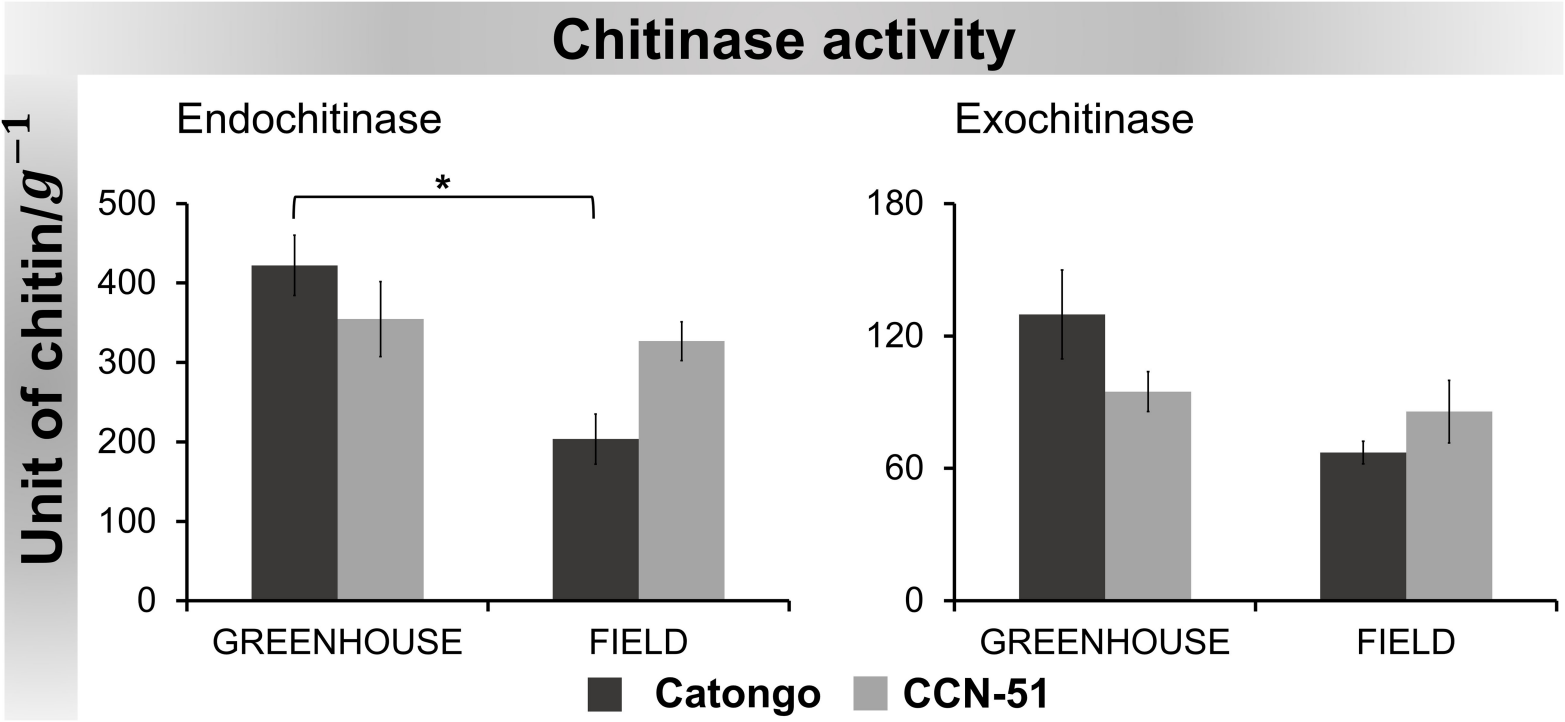
Chitinase activity in apoplastic washing fluid from *T. cacao* genotypes. The points represent the means of the triplicates of the analyses of each variety. * Significance of p < 0.05 by ANOVA followed by comparison using the Tukey test (p < 0.01 and p < 0.05). Bars correspond to standard errors of the means.

Chitinase activity maintained similar behavior for both substrates in all samples, with higher activity detected in Catongo GH and lower activity in Catongo FD. A statistically significant difference (p < 0.05) was observed between these samples when evaluated with the substrate for endochitinase detection. Catongo GH showed activity approximately two times greater than Catongo FD (**Figure 8**).

**Figure 9:**
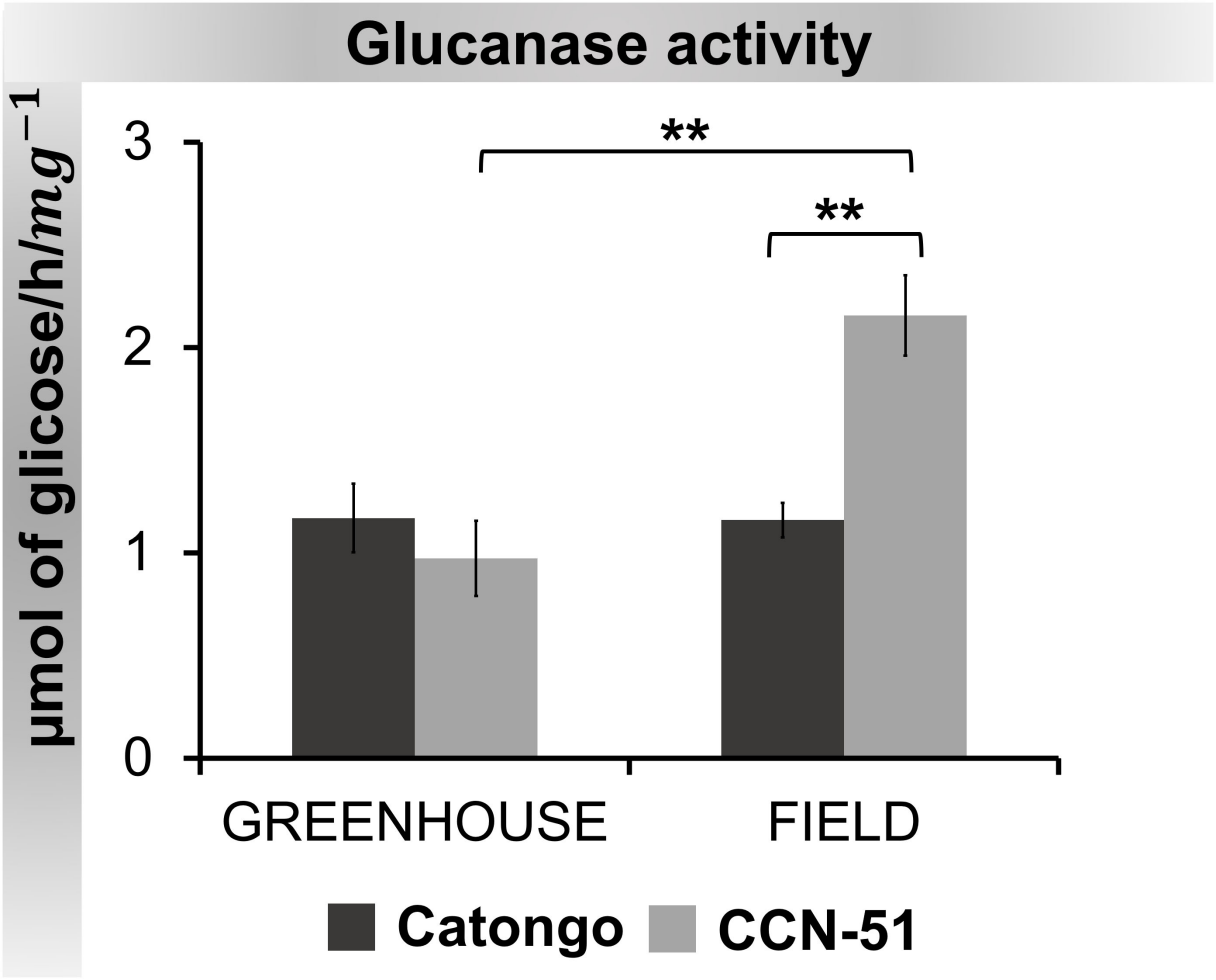
β-1,3-Glucanase activity in apoplastic washing fluid from *T. cacao* genotypes. The points represent the means of the triplicates of the analyzes of each variety. ** Significance of p < 0,01 by ANOVA followed by comparison using the Tukey test (p < 0.01 and p < 0.05). Bars correspond to standard errors of the means.

The activity of the enzyme β-1,3-glucanase was higher in CCN-51 FD compared to the other samples. Activity was significantly (p < 0.01) higher in CCN-51 FD, both compared to the GH condition and the Catongo FD sample (**Figure 9**).

**Figure 10:**
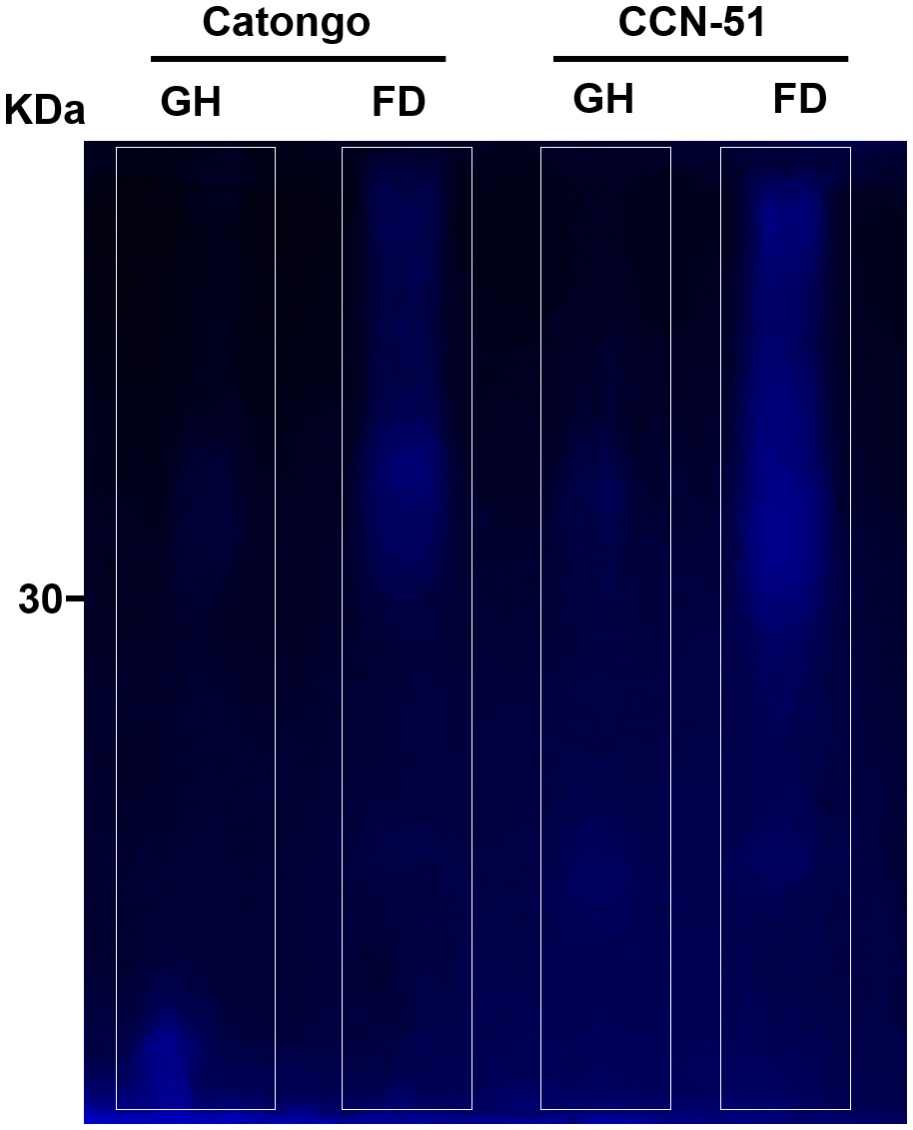
Proteases activity in apoplastic washing fluid from *T. cacao* genotypes. Zymogram with protease activity in apoplastic washing fluid of *T. cacao* of the Catongo (susceptible) and CCN-51 (resistant) genotypes of asymptomatic plants grown in a greenhouse (GH) and plants at the green witch’s broom stage grown under conditions of field (FD). kDa, Molecular weight.

To evaluate protease activity in the crude AWF extract of *T. cacao* genotypes, zymography was performed on a polyacrylamide gel. The gel image (**Figure 10**) shows clear regions (highlighted with white outlines) where the proteases present in AWF have degraded the substrates used in the packaging gel, confirming their activity and location in the gel. The clear and continuous regions throughout the gel can be noted mainly above 30 kDa.

#### 3.8 Microorganism proteins in the AWF of *T. cacao*

Microorganism proteins were identified in the AWF of the Catongo and CCN-51 genotypes of *T. cacao*. Most of the proteins belong to the fungus *M. perniciosa*. Twenty-three proteins were identified in the Catongo and CCN-51 FD genotypes, with eight proteins being exclusive to the susceptible genotype (Catongo) and 10 being exclusive to the resistant genotype (CCN-51). Five *M. perniciosa* proteins were common to both genotypes (**Tables 1**, **2**).

**Table 1:** Proteins from microorganisms identified in apoplastic washing fluid of the Catongo GH and FD genotype.

	Accession	Species	Protein ID	Protein_MW (Da)	Protein_pI
Catongo GH					
**1**	A0A5N5CX20	*Lasiodiplodia theobromae*	Elongation factor 1-alpha	50,346	9.39
**2**	E2LT56	*Moniliophthora perniciosa*	Ribose-phosphate diphosphokinase	25,506.1	6.08
**3**	E2LP61	*Moniliophthora perniciosa*	ArgoN domain-containing protein (fragment)	17,483.8	8.8
**4**	Q756G2.2	*Ashbya gossypii*	RecName: Full=Probable E3 ubiquitin-protein ligase TOM1	374,219.9	4.99
**5**	Q9UT10.1	*Schizosaccharomyces pombe*	RecName: Full=Uncharacterized GTP-binding protein P8A3.05	78,337	9.19
Catongo FD					
**1**	E2LZF6	*Moniliophthora perniciosa*	Rhizoctonia solani AG1-IB WGS project CAOJ00000000 data, isolate 7/3/14, contig (fragment)	31,159.7	8.8
**2**	E2L970	*Moniliophthora perniciosa*	Protein kinase domain-containing protein (fragment)	26,816.9	5.82
**3**	E2LKI3	*Moniliophthora perniciosa*	Peroxisomal membrane protein 11B	23,070.2	10.08
**4**	E2LMQ4	*Moniliophthora perniciosa*	CAC1F_C domain-containing protein (fragment)	30,372.4	6.17
**5**	E2LWM7	*Moniliophthora perniciosa*	Gluconate transport inducer 1/Pac2 (fragment)	32,475.9	9.28
**6**	E2LLF1	*Moniliophthora perniciosa*	NAD(P)-binding protein (fragment)	15,120.1	5.12
**7**	E2L833	*Moniliophthora perniciosa*	DUF2428 domain-containing protein (fragment)	17,704.9	7.04
**8**	E2M402	*Moniliophthora perniciosa*	Btz domain-containing protein (fragment)	19,962.6	3.88
**9**	E2LQ59	*Moniliophthora perniciosa*	DUF4238 domain-containing protein (fragment)	39,094.1	6.11
**10**	E2LGU4	*Moniliophthora perniciosa*	PFK domain-containing protein (fragment)	20,282.8	8.23
**11**	E2LD68	*Moniliophthora perniciosa*	fn3_5 domain-containing protein	37,933.7	4.43
**12**	E2L3A9	*Moniliophthora perniciosa*	Peptidase A2 domain-containing protein (fragment)	21,452.5	9.36
**13**	E2LQ59	*Moniliophthora perniciosa*	DUF4238 domain-containing protein (fragment)	39,094.1	6.11
**14**	A0A5N5DBM4	*Lasiodiplodia theobromae*	Protein transport protein SEC13	33,030.2	5.86
**15**	Q756G2.2	*Ashbya gossypii*	RecName: Full=Probable E3 ubiquitin-protein ligase TOM1	374,219.9	4.99
**16**	O74465.2	*Schizosaccharomyces pombe*	RecName: Full=Helicase required for RNAi-mediated heterochromatin assembly 1	115,136.9	6.12
**17**	O74762.1	*Schizosaccharomyces pombe*	RecName: Full=26S proteasome regulatory subunit rpn2	107,730.9	4.93
**18**	A0A0S6XHH0.1	*Fungal* sp	RecName: Full=FR901469 synthetase	1,721,677.5	5.3
**19**	Q6BKW6.2	*Debaryomyces hansenii*	RecName: Full=Protoheme IX farnesyltransferase, mitochondrial	51,651.1	9.91

**Table 2:** Proteins from microorganisms identified in apoplastic washing fluid of the CCN-51 GH and FD genotypes.

	Accession	Species	Protein ID	Protein_MW (Da)	Protein_pI
CCN-51 GH					
**1**	Q6FPW3.1	*Candida glabrata*	RecName: Full=Glyceraldehyde-3-phosphate dehydrogenase 1	36,089.3	5.25
**2**	E2LT56	*Moniliophthora perniciosa*	Ribose-phosphate diphosphokinase	25,506.1	6.08
**3**	Q6FM42.1	*Candida glabrata*	RecName: Full=Peroxisomal targeting signal receptor	66,872.2	4.88
**4**	Q6CG48.1	*Yarrowia lipolytica*	RecName: Full=Nuclear distribution protein PAC1	48,693.1	7.37
**5**	Q756G2.2	*Ashbya gossypii*	RecName: Full=Probable E3 ubiquitin-protein ligase TOM1	374,219.9	4.99
**6**	P15442.3	*Saccharomyces cerevisiae*	RecName: Full=eIF-2-alpha kinase GCN2	190,589.3	5.98
CCN-51 FD					
**1**	E2LVC0	*Moniliophthora perniciosa*	Aminotran_1_2 domain-containing protein (fragment)	44,596.5	4.89
**2**	E2L970	*Moniliophthora perniciosa*	Protein kinase domain-containing protein (fragment)	26,816.9	5.82
**3**	E2LZF6	*Moniliophthora perniciosa*	Rhizoctonia solani AG1-IB WGS project CAOJ00000000 data, isolate 7/3/14, contig (fragment)	31,159.7	8.8
**4**	E2L603	*Moniliophthora perniciosa*	G_PROTEIN_RECEP_F1_2 domain-containing protein (fragment)	20,709	9.66
**5**	E2LMQ4	*Moniliophthora perniciosa*	CAC1F_C domain-containing protein (fragment)	30,372.4	6.17
**6**	E2LXQ7	*Moniliophthora perniciosa*	Cytochrome P450	35,812.5	9.77
**7**	E2LWM7	*Moniliophthora perniciosa*	Gluconate transport inducer 1/Pac2 (fragment)	32,475.9	9.28
**8**	E2LEU9	*Moniliophthora perniciosa*	40S ribosomal protein S17 (fragment)	23,822.1	9.83
**9**	E2LMX7	*Moniliophthora perniciosa*	Translation elongation factor 1-alpha	33,561.9	9.32
**10**	E2LCF0	*Moniliophthora perniciosa*	Kinase	13,470.3	6.83
**11**	E2LFU0	*Moniliophthora perniciosa*	FYR N-terminal domain-containing protein	22,653.3	7.94
**12**	E2LD68	*Moniliophthora perniciosa*	fn3_5 domain-containing protein	37,933.7	4.43
**13**	E2LGJ7	*Moniliophthora perniciosa*	FGGY_C domain-containing protein	28,311.4	6.19
**14**	E2L9L1	*Moniliophthora perniciosa*	Cyclin-domain-containing protein	12,219.8	8.08
**15**	E2LEA8	*Moniliophthora perniciosa*	DUF3475 domain-containing protein	18,919.1	5.49
**16**	A0A5N5DN88	*Lasiodiplodia theobromae*	Uncharacterized protein	52,449.6	5.56
**17**	Q756G2.2	*Ashbya gossypii*	RecName: Full=Probable E3 ubiquitin-protein ligase TOM1	374,219.9	4.99
**18**	P47155.1	*Saccharomyces cerevisiae*	RecName: Full=Protein ILM1	23,758.4	4.35
**19**	P07754.1	*Aspergillus nidulans*	RecName: Full=Alcohol dehydrogenase 3	37,524.8	6.62
**20**	Q9USW2.1	*Schizosaccharomyces pombe*	RecName: Full=Inheritance of peroxisomes protein 2	55,425.6	8.39

Interestingly, in Catongo GH, proteins from the filamentous fungus *M. perniciosa* were found, as well as from the fungi *Lasiodiplodia theobromae* and *Ashbya gossypi* and the yeast *Schizosaccharomyces pombe*. In the CCN-51 GH genotype, a protein from *M. perniciosa* was also found, in addition to proteins from *Candida glabrata*, *Yarrowia lipolytica*, *A. gossypi* and *Saccharomyces cerevisiae*. In Catongo FD, proteins from *M. perniciosa*, *L. theobromae*, *A. gossypi*, *S. pombe*, and *Debaryomyces hansenii* were found. Proteins from *M. perniciosa*, *L. theobromae*, *A. gossypi*, *S. pombe*, as well as *Aspergillus nidulans* and *S. cerevisiae* were also found in CCN-51 FD (**Tables 1**, **2**).

## 4 Discussion

### 4.1 The AWF extraction step improves the identification of proteins secreted in *T. cacao* leaves

Obtaining a good yield of apoplast proteins, which can be soluble in AWF or linked to the cell wall, is still a challenge, due to the difficulties of avoiding disruption of the cell wall and membrane. As a result, they are still poorly characterized (Delaunois et al., 2014).

The technique for obtaining apoplastic proteins, which initially involves the extraction of AWF, demonstrated a high protein yield in samples of *T. cacao* leaves, despite presenting cytoplasmic contamination of 56% (**Figure 3**). The number of apoplastic proteins identified (27) was greater from AWF extraction compared to obtaining apoplastic proteins from TE (19). For the recovery of apoplastic proteins from the TE of *T. cacao* leaves (**Figure 3**), cytoplasmic contamination of 87% was observed, corresponding to 132 proteins out of a total of 151, due to the leaf maceration process.

The total number of proteins recovered is reduced using the optimized AWF extraction technique from *T. cacao* leaves (62 proteins), compared to the number of proteins in the TE sample (151 proteins). However, it is important to highlight that the concentration of proteins in the apoplast is lower compared to those present in the internal space of the cells (Delaunois et al., 2014), additionally obtaining AWF also reduces cytoplasmic contamination compared to TE samples. The centrifugal force used in AWF extraction is essential to reduce this contamination, and the centrifugation-infiltration method that was used in this work has already been adopted in several works (Soares et al., 2007; Delannoy et al., 2008; Pirovani et al., 2008; Pechanova et al., 2010; de Oliveira et al., 2024), being adapted for each species and enabling better investigation within the area of subcellular proteomics.

### 4.2 The apoplast is rich in defense and stress proteins

Most of the proteins identified in the AWF of *T. cacao* genotypes are related to defense and stress, with emphasis on pathogenesis-related proteins (PR-proteins).

PR-proteins make up an important class of proteins that play a crucial role in the plant defense response against pathogens. The production of these proteins is increased when the plant is under stress, and they perform several functions, ranging from directly inhibiting the growth of pathogens to strengthening the plant’s defenses (Sels et al., 2008; Sudisha et al., 2012; dos Santos and Franco, 2023).

Currently, 19 families of PRs have been described (dos Santos and Franco, 2023), among which we identified representatives of the families PR-3, PR-4, PR-5, PR-9, and PR-14. Based on the analysis of cacao transcripts using public data carried out in a previous work (Teixeira et al., 2014), some transcripts belonging to apoplastic PR-proteins were up-accumulated in infected plants compared to healthy *T. cacao* (Forastero) plants used by Teixeira et al. (2014), as members of PR-3, PR-4, PR-5, PR-9, and PR-14 (**Figure 4**). This corroborates findings in the present work, due to the importance that PR-proteins have in host defense.

Proteins from the PR-3 family (chitinases) were detected only in the resistant genotype (CCN-51), with most representatives showing a greater accumulation in green brooms (FD) (**Figure 1**). Recently, chitinases (EC 3.2.1.14) were identified in the AWF of *T. cacao* only in the FD-grown CCN-51 genotype (de Oliveira et al., 2024). This corroborates the data revealed on PR-3 accumulation in CCN-51 FD (**Figure 7**). Although not identified by proteomic analysis, the protein was also accumulated in the Catongo GH and FD genotypes (susceptible) and verified by Western blot (**Figure 7**). A previous study demonstrated that the Catongo genotype contained a greater amount of upregulated defense and stress-related proteins, such as an acid chitinase isoform, at 45 days after inoculation with *M. perniciosa*, compared to the downregulated proteins (dos Santos et al., 2020). The genotype of *T. cacao* resistant to WBD (TSH1188) showed upregulated chitinase isoforms 42 h after inoculation and 45 days after inoculation with *M. perniciosa* (dos Santos et al., 2020).

Chitinases play an effective role in breaking down chitin, an essential component in the constitution of the fungal cell wall. One of the main functions of chitinases in plants is associated with the defense response against pathogenic fungi, but they can also have antibacterial and antiviral actions (dos Santos and Franco, 2023). In the *T. cacao* apoplast of the CCN-51 genotype (resistant), chitinases were identified, represented in cluster 1 and are involved in the defense response (**Figure 6**). According to the network of interactions, these proteins have a high betweenness value and can be considered bottleneck proteins. Bottleneck proteins play an important role in the regulation and control of metabolic pathways and/or cell signaling and are, therefore, essential in the communication and integration of different parts of the network (Nithya et al., 2023).

Chitinases are important in cell signaling, due to their chitinolytic activity and the formation of oligosaccharides, which act as elicitor molecules that are recognized by the host plant, favoring a cascade of signals (Kaur et al., 2022; dos Santos and Franco, 2023). Extracellular chitinases can have direct antimicrobial action by the degradation of invading hyphae (Stangarlin and Pascholati, 2000) and can be acidic or basic (Vaghela et al., 2022).

PR-4 was identified in both *T. cacao* genotypes, being up-accumulated in the FD condition (green witch’s broom stage) (**Figure 1**). When comparing protein accumulation between genotypes under the same condition, PR-4 was up-accumulated in the CCN-51 FD (resistant) genotype and down-accumulated in Catongo FD (susceptible) (**Figure 2**). PR-4 can act directly on the pathogen’s cell wall due to its ability to bind to chitin and may present chitinolytic activity (Ali et al., 2018; Butassi et al., 2022). Recently, this protein showed increased abundance in the apoplast of peach plants inoculated with the fungus *Taphrina deformans* (Butassi et al., 2022). The antifungal activity of PR-4 was proven in wheat against fungi of the genus *Fusarium* (Caruso et al., 1996), in transgenic grapevines in response to powdery mildew disease (Dai et al., 2016) and in response to abiotic stress in rice (Wang et al., 2011).

The PR-4 protein identified in Catongo is represented in cluster 1, involved in the carbohydrate metabolic process. In the CCN-51 genotype, it is represented in cluster 1 and has a high betweenness value and can be considered a bottleneck protein (**Figure 6**).

Thaumatin-like protein (PR-5) was detected in the Catongo (susceptible) and CCN-51 (resistant) genotypes exclusively in the FD condition (**Figure 1**). The PR-5 protein is recognized for its role in plants’ defense response against a variety of pathogens. It can accumulate in the apoplast or vacuole and act directly as an antifungal agent, inhibiting the growth and spread of invasive fungi (Stintzi et al., 1993; de Jesús-Pires et al., 2020; Manghwar and Hussain, 2021). Previous studies have demonstrated that the PR-5 protein delayed the disease caused by *Phytophthora infestans* in potato (Liu et al., 1994). In garlic, PR-5 genes are involved in defense against *Fusarium* infection (Anisimova et al., 2021). Silencing of a *PR-5* gene in the wheat apoplast leads to compromised resistance against the pathogen *Puccinia triticina* (Zhang et al., 2018). Recently, an osmotin (PR-5) was identified in the apoplast of the WBD-resistant genotype of *T. cacao* (de Oliveira et al., 2024). The Catongo thaumatin-like protein is represented in cluster 1, being involved in the carbohydrate metabolic process (**Figure 6**). In CCN-51, it is involved in the defense response (cluster 1) representing a bottleneck protein within the network (**Figure 6**). Accordingly, it is suggested that this protein plays a crucial role in immune responses in *T. cacao*, contributing to the reinforcement and tentative resistance of *T. cacao* to pathogenic threats.

Peroxidases (PR-9) were detected in both genotypes under both growth conditions. Peroxidase 70 was exclusively detected in Catongo FD, peroxidase A2 was up-accumulated in Catongo and CCN-51 GH, and peroxidase 10 was exclusively detected in CCN-51 GH. The expression of two peroxidases was increased in the apoplast of barley infected by the pathogen *Pyrenophora teres* f*. teres* (Hassett et al., 2020). This protein plays an important role against pathogens in the initial stages of infection, acting as an antioxidant enzyme, and also actively participates in cell wall synthesis and remodeling (Hiraga et al., 2001; dos Santos and Franco, 2023). In CCN-51, peroxidase 10 is represented in cluster 4 (**Figure 6**), related to the response to oxidative stress. According to the findings in the present work, the production of peroxidases may be induced in infected FD genotypes. Because some PR-proteins can be produced constitutively (van Loon et al., 2006), it is likely that this is occurring in GH genotypes. Therefore, in normal situations, plants may express peroxidases to manage natural ROS production.

The PR-14 protein (nsLTPs) was detected exclusively in the Catongo GH genotype. PR-14 facilitates the transfer of lipids and has antifungal and antibacterial activity, by acting on the permeability of the fungal/bacterial membrane (Van Loon and Van Strien, 1999; Hoffmann-Sommergruber, 2002; Gorjanović et al., 2005; Patkar and Chattoo, 2006). This protein was represented in cluster 8, involved in lipid transport (**Figure 6**), but the exact biological role remains to be determined.

### 4.3 Down-accumulated proteins in the apoplastome in *T. cacao* genotypes suggest sequestration of defense pathways in FD genotypes by *M. perniciosa*

Asymptomatic *T. cacao* plants grown in the GH had a greater number of proteins identified in the apoplastome, both in comparison within and between genotypes in relation to green witches’ broom plants grown in the FD. This may be related to the modulation of the defense system (Nishad et al., 2020). It can be inferred that the defense system of FD plants was previously turned off due to more dynamic interactions with the environment and that *M. perniciosa* infection favors the reduction of prolonged defense.

In a previous work, it was proven that 45 days after inoculation with *M. perniciosa* in the Catongo (susceptible) and TSH1188 (resistant to WBD) genotypes, the accumulation of proteins related to stress and defense is reduced in Catongo, whereas, in TSH1188, it was significantly increased (dos Santos et al., 2020). The period of onset and development of WBD in the FD plants used in the present work is unknown. However, presumably more than 45 days had already passed since the onset of the disease, which may explain the reduction in protein accumulation in the genotypes of infected plants (FD).

A cysteine-rich secretory protein showed increased accumulation in the Catongo (susceptible) and CCN-51 (resistant) GH genotypes but decreased in CCN-51 FD and was not detected in Catongo FD. The cysteine-rich repeat protein is apoplastic and plays a fundamental role in plant defense against phytopathogens. A cysteine-rich repeat protein from wheat was proven to be effective against the phytopathogens *Rhizoctonia cerealis* and *Bipolaris sorokiniana*, through its proven antifungal activity in inhibiting mycelium growth, as well as regulating the expression of pathogenesis-related genes such as of β-1,3-glucanase and chitinases (Guo et al., 2020). A cotton cysteine-rich repeat protein acted on the stability of a chitinase, protecting it from cleavage by *Verticillium dahliae* protease. Overexpression of the protein increased resistance against the cotton fungal phytopathogen (Han et al., 2019). The Catongo cysteine-rich secretory protein is represented in cluster 2 and in CCN-51 in cluster 3, being involved in the steroid biosynthetic process in both genotypes (**Figure 6**). It is likely that this protein acts in the production of important compounds in the stress response of *T. cacao.* The reduced accumulation of cysteine-rich repeat protein in *T. cacao* FD (green witch broom stage) genotypes may have been mediated by the strong inhibitory activity of the fungus *M. perniciosa*, unlike what occurred in *T. cacao* GH (asymptomatic grown in a GH) genotypes.

The protein SOD was detected in both genotypes only in the GH condition. SOD plays a fundamental role in antioxidant defense in plants due to the increase in reactive oxygen species (ROS) (Mase and Tsukagoshi, 2021). SOD acts as the first line of defense against oxygen free radicals in less toxic forms, such as hydrogen peroxide (H_2_O_2_) and oxygen (O_2_), reducing oxidative damage (Qi et al., 2017; Wang et al., 2018). The main type of SOD found in the apoplast is copper-zinc (Cu-Zn SOD) (Alscher et al., 2002). The SOD protein identified in Catongo (susceptible) GH is represented in cluster 3 and may be involved in the generation of precursor metabolites and energy (**Figure 6**). This protein is classified according to the biological network as a “hub” protein, where it plays an important role in the transmission of signals, the integration of metabolic pathways, and the regulation of biological processes (Nithya et al., 2023).

Increased SOD accumulation may be associated with increased ROS, especially in response to biotic stress (Jwa and Hwang, 2017; Wang et al., 2018). The decrease in SOD in FD genotypes (green witch broom stage) in the present work may be an effect of the plant’s response to infection, where manipulation of antioxidant defenses may be a strategy used by the pathogen *M. perniciosa* to facilitate disease progression. Therefore, the fungus possibly interferes with the defense mechanisms of *T. cacao*, reducing the effectiveness of antioxidant enzymes such as SOD, which could result in greater oxidative damage and benefit the pathogen in the plant.

### 4.4 The activity of enzymes occurring in the *T. cacao* apoplast

Some PR-proteins that have enzymatic action, such as PR-3 and PR-2, were evaluated in the present study.

The chitinases evaluated, although they demonstrated a similar pattern of activity in all samples, exhibited higher endochitinase activity compared to the exochitinase activity (**Figure 8**). Plant chitinases are generally endochitinases with a molecular weight of 25 kDa to 36 kDa (Hoffmann-Sommergruber, 2002; Hamid et al., 2013; Vaghela et al., 2022) and are active in plant self-defense in response to phytopathogen attack (Vaghela et al., 2022).

For endochitinase (EC 3.2.1.14) activity, a statistically significant difference was observed between samples of the susceptible genotype (Catongo GH and FD) (**Figure 8**). The activity in the Catongo GH genotype was significantly higher than in the FD sample. Although proteomic data did not reveal the accumulation of chitinase in Catongo GH, it was possible to measure its ability to catalyze reactions in this genotype. The use of crude AWF extract with proteins in the native state may have favored its dosage in GH samples (asymptomatic plants grown in a GH).

When evaluating PR-2 activity, we observed statistically significant increases between CCN-51 FD and GH and between CCN-51 FD and Catongo FD, approximately twice as high (**Figure 9**). Increased PR-2 activity after infection was reported in rice infected by *Magnaporthe oryzae* (Wang et al., 2021), in chickpea AWF infected by *Ascochyta rabiei* (Hanselle and Barz, 2001), and in resistant cultivars of wheat infected by *Diuraphis noxia* (Van Der Westhuizen et al., 1998).

PR-2 (EC 3.2.1.39) has beta-1,3-glucanase activity, an enzyme that breaks beta-1,3-glucan bonds, an important component in the cell wall of many fungi (Hoffmann-Sommergruber, 2002). Therefore, beta-1,3-glucanases play a crucial role in the defense response of plants against fungal pathogens, because the breakdown of beta-1,3-glucan compromises the integrity of the fungal cell wall (Balasubramanian et al., 2012; dos Santos and Franco, 2023). This can lead to the death of the fungus or at least weaken its ability to invade and colonize plant tissue.

The apoplastic AWF of *T. cacao* genotypes also revealed a high protease activity through the zymogram gel (**Figure 10**), confirming the activity of proteases, such as the subtilases identified in the present work: protease secreted by CO_2_ response and similar proteases to subtilisin (XP_017981649.1, XP_007017870.2, XP_007018543.2 and XP_007017195.2). Subtilases are very numerous in plants and can be directed to the cell wall and contribute to wall modification, cell signaling, and response to biotic and abiotic stress (Figueiredo et al., 2014; Schaller et al., 2018). A subtilase gene in cotton is involved in the plant’s defense against the pathogen *Verticillium dahliae* (Duan et al., 2016). The identification of subtilases in AWF has already been reported in coffee leaves (Guerra-Guimarães et al., 2015), *Arabidopsis thaliana* leaves (Jiang et al., 2023) and leaves of the Catongo and CCN-51 genotypes of *T. cacao* (de Oliveira et al., 2024). The transcript belonging to two protein isoforms were up-accumulated (lcl_NC_030857.1_mrna_XM_018126160.1_26400 and lcl_NC_030857.1_mrna_XM_007018481.2_26945) (**Figure 4**). The activity of these proteases in the *T. cacao* apoplast, together with others identified and shown in **Figures 1**, **2**, may be important in the infection process by modulating the plant’s defense responses.

### 4.5 Peptides from microorganisms are detected in the apoplast of *T. cacao* genotypes

A total of 40 proteins from microorganisms were identified in the AWF of *T. cacao* genotypes, 23 of which belonged to the fungus *M. perniciosa* present in the FD genotype samples (plants in the green witch’s broom stage) (**Tables 1**, **2**). Some functions of these proteins are associated with biosynthetic processes, protein transport, and proteolysis. The FD genotypes were under a high level of stress, evident with the branches at the green witch’s broom stage used in AWF extraction, and, in this context, it was predicted that the fungus *M. perniciosa* would secrete molecules in the *T. cacao* apoplast in order to interfere with the plant defense (Fiorin et al., 2018).

It is known that the microbial community can exert a symbiotic relationship with plants, resulting from coevolution between species (Pathak et al., 2022). This relationship is possible because their genomes were shaped to survive together, so they stand out for having characteristics that prevent the recognition of microorganisms by the plants’ immune systems (Khare et al., 2018; Pathak et al., 2022).

A protein belonging to an unidentified fungal species (ID: A0A0S6XHH0.1) was found in the Catongo GH sample (**Table 1**). This protein’s beta-1,3-glucan inhibitory activity has already been established by *in vitro* and *in vivo* studies, showing antifungal activity against the fungi *Candida albicans* and *Aspergillus fumigatus* (Fujie et al., 2000a, Fujie et al., 2000b). We suggest that the fungus has a beneficial relationship in Catongo against possible invaders. Additional studies are essential for a more thorough understanding of its action.

In a previous work, the diversity of endophytic fungi in *T. cacao* (Rubini et al., 2005) was proven, including the fungus *Lasiodiplodia theobromae*. This corroborates our data from this study, where peptides from *L. theobromae* were identified. Interestingly, *L. theobromae* peptides were detected in both GH and FD plants. *L. theobromae* is a pathogenic necrotrophic fungus important to several crops, including *T. cacao* (Pereira et al., 2006; de Oliveira et al., 2013). Under controlled conditions, the first symptoms can be visible within 2 weeks after inoculation, such as wilting and death within 10 days (Moreira-Morrillo et al., 2021). Because the GH genotypes (asymptomatic plants grown in a GH) had no visible symptoms of any of the fungi mentioned, it is necessary to perform more comprehensive investigations, using other methods, in order to more precisely elucidate the identification of these peptides and/or functions in the plant, such as metagenome sequencing.

Yeast peptides were also found in both genotypes. Endophytic yeasts have been associated with several plant species: *Saccharomyces cerevisiae*, considered endophytic of *Ficus carica* (Ling et al., 2020); and *Debaryomyces hansenii*, endophytic of wheat (Wachowska et al., 2018) and rice (Tantirungkij et al., 2015), all identified in the apoplast of *T. cacao*. Although endophytes may play a role in increasing growth, acquiring nutrients and strengthening tolerance to stress in plants (Ling et al., 2020), additional studies are needed. Such studies would offer a more comprehensive understanding of the possible symbiotic relationship between these microorganisms and *T. cacao*, clarifying the benefits for the host plant.

## 5 Conclusion

In this study, we report the dynamics and differences of the apoplast proteome between Catongo (WBD-susceptible) and CCN-51 (WBD-resistant) *T. cacao* genotypes, asymptomatic, and infected by the destructive *pathogen M. perniciosa*. Proteins detected in the apoplast are related to the plant’s defense response; in addition to the enzymatic profile of chitinases, β-1,3-glucanases, and proteases present in AWF, we demonstrate the great importance of the *T. cacao* apoplast during the plant–pathogen interaction. A possible modulation and suppression of the *T. cacao* defense system promoted by *M. perniciosa* can be suggested, based on the protein profile of the FD genotypes with a significant number of down-accumulated defense proteins. A symbiotic relationship between microorganisms and *T. cacao* has been proposed; however, more in-depth studies should be carried out with the addition of different methodologies to confirm endophytes and their possible benefits for *T. cacao*. The *T. cacao* apoplast reveals important responses in the molecular battle *against M. perniciosa* and the crucial phase of the infection, biotrophic, can be understood in the light of the apoplastome. Target proteins identified here can be functionally explored in future studies with biotechnological applications in order to control WBD.

## Data availability statement

The datasets presented in this study can be found in online repositories. The names of the repository/repositories and accession number(s) can be found in the article/**Supplementary Material**.

## Author contributions

IdO: Data curation, Formal analysis, Investigation, Methodology, Writing – original draft. SA: Investigation, Methodology, Writing – original draft. MF: Conceptualization, Investigation, Methodology, Software, Writing – original draft. AS: Data curation, Software, Writing – original draft. KF: Investigation, Methodology, Writing – original draft. EA: Formal analysis, Investigation, Methodology, Writing – original draft. IM-O: Conceptualization, Methodology, Software, Writing – original draft. JM: Software, Writing – original draft. EC: Software, Writing – original draft. KG: Investigation, Methodology, Supervision, Writing – original draft. CP: Conceptualization, Funding acquisition, Project administration, Resources, Supervision, Visualization, Writing – original draft.
