# Supplementary material for: Apoplastomes of contrasting cacao genotypes to witches’ broom disease reveals differential accumulation of PR proteins
Source: Front Plant Sci. 2024 May 16;15:1387153. doi: 10.3389/fpls.2024.1387153 (PMC11137319; doi:10.3389/fpls.2024.1387153)
Supplement: Supplementary file 1 [file DataSheet_1.pdf]

Supplementary Material

Supplementary Figure 1

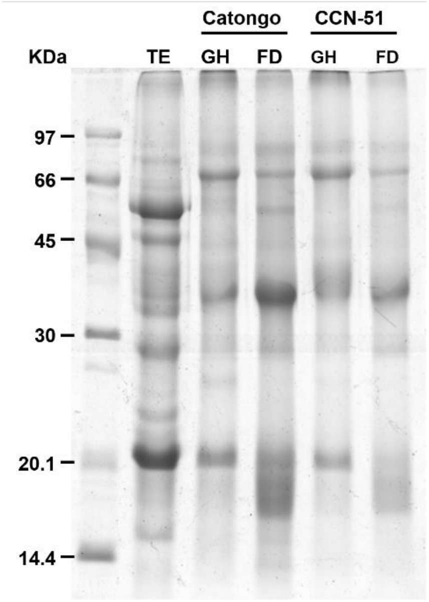

Supplementary Figure 1 Protein profile of apoplastic washing fluid of *T. cacao* genotypes Catongo (susceptible) and CCN-51 (resistant) according to SDS-PAGE.; TE: CCN-51 genotype total extract; GH: proteins from apoplastic washing fluid from plants kept in a greenhouse; FD: apoplastic washing fluid proteins from green witches' broom leaves collected under field conditions. kDa: Molecular Weight Marker.

Supplementary Figure 2

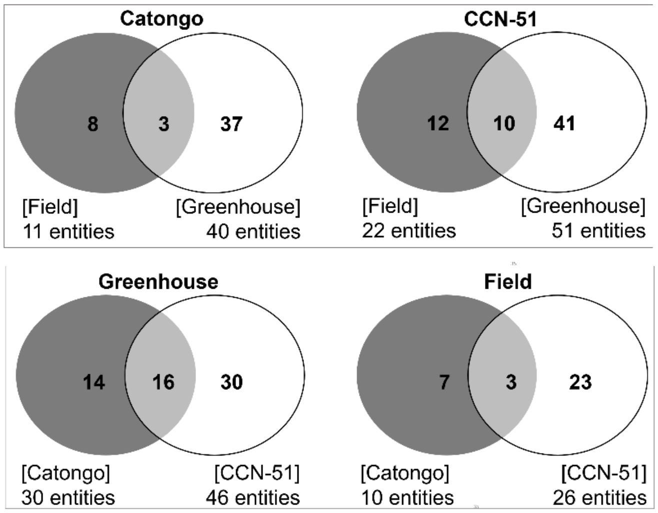

Supplementary Figure 2 Venn diagram representing the unique and common total proteins ($p \leq 0.05$ and Fold change ≥ 1.5) identified in the apoplastic washing fluid of *T. cacao* genotypes Catongo (susceptible) and CCN-51 (resistant) from asymptomatic plants grown in a greenhouse and of green witches' broom stage plants grown under field conditions.

Supplementary Figure 3

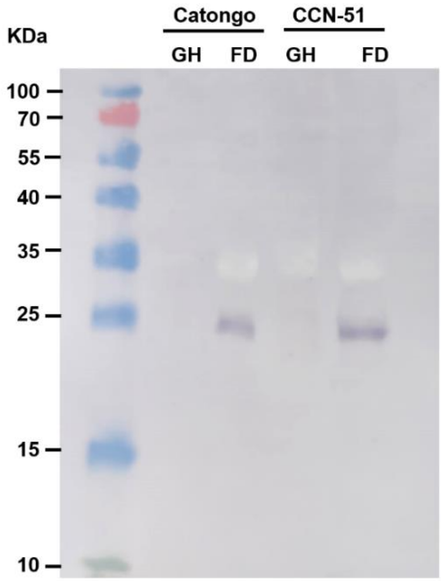

Supplementary Figure 3 PR-3 protein accumulation in samples of apoplastic washing fluid from *T. cacao* genotypes Catongo (susceptible) and CCN-51 (resistant) from asymptomatic plants grown in a greenhouse (GH) and of green witches' broom stage plants grown under field conditions (FD).
